# Productivity trade-off with different water regimes and genotypes of rice under non-puddled conditions in Eastern India

**DOI:** 10.1016/j.fcr.2017.10.007

**Published:** 2018-06-01

**Authors:** Ipsita Kar, Sudhir-Yadav, Amit Mishra, Basudev Behera, Chandramani Khanda, Virender Kumar, Ashok Kumar

**Affiliations:** a Orissa University of Agriculture and Technology, Bhubaneshwar, Odisha, India; b International Rice Research Institute, Los Baños, Philippines

**Keywords:** Direct seeding, Irrigation scheduling, Water saving, Hybrids

## Abstract

Increasing farm labor scarcity and depletion of natural resources such as water are posing a major threat to the sustainability of traditional puddled transplanted rice (PTR) farming in Eastern India. Dry-seeded rice (DSR) or non-puddled transplanted rice (NPTR) could be used as an alternative to PTR. To understand the trade-off with different water management and rice genotypes under non-puddled conditions, a field experiment was conducted during 2014–2015 on a sandy clay loam soil of Bhubaneswar, Odisha. The treatments for water regimes were based on soil water tension (no stress, 10 kPa, and 40 kPa) at 15-cm soil depth and the cultivars used in the study were inbreds (Lalat and Sahbhagi Dhan) and hybrids (Arize^®^ 6129, 6444, and US 323).

In both years, rice yields were higher in the dry season than in the wet season. However, both establishment methods performed similarly in all the seasons. With an increase in water stress, there was a significant decline in yield and yield attributes in the dry season. Irrigation input in the dry season was roughly more than double that in the wet season. Irrigation input was relatively higher in DSR than in NPTR in all the seasons, which might be because of an extra irrigation required for DSR crop establishment than for transplanting in non-puddled conditions where watering is done only for ease of transplanting. There was irrigation saving of 25% and 58% in 10 kPa and 40 kPa, respectively, compared to no stress in the dry season. A consistent trend of an increase in irrigation water productivity (WP_I_) and input water productivity (WP_I+R_) was observed with an increase in stress. Arize^®^ 6444 produced the highest grain yield, irrigation and input water productivity, and its performance was also better in terms of tiller density, LAI, and biomass. Our findings highlight the potential of hybrids compared with inbreds and their performance under DSR was found to be superior. Even though the yield in no stress was slightly higher than in 10and kPa, the irrigation water savings in 10 kPa were distinctly significant.

## Highlights

• Rice yield was not affected by planting method (dry seeding or non-puddled transplanting).
• Irrigation input was relatively higher in DSR than in NPTR in all the seasons.
• Increase in irrigation water productivity and input water productivity with alternate wetting and drying.
• High the potential of hybrids for non-puddled system.

## 1 Introduction

Rice is an important staple food in Asia, covering 85% of the total world rice area (FAI, 2009, IRRI, 2006). In Asia, rice is grown on 143 Mha, out of which 44 Mha are grown in India, contributing about 106.7 million tons of grain production, out of which dry-season (*rabi)* rice adds 15.2 million tons while the remaining 91.5 million tons come from wet-season (*kharif)* rice (GOI, 2016a). The stagnation in the productivity of rice and depletion of natural resources in Northwest India (Ladha et al., 2003, Pathak et al., 2003, Rodell et al., 2009) for the past decade have impelled the country to put extra efforts into increasing production and productivity in Eastern India. A major part of Eastern India receives about 1268 mm of rainfall (Kumar et al., 2010), therefore has favorable conditions for rice production. However, the present productivity of rice in Eastern India is low of ∼2 t ha^−1^ (GOI, 2016b, Sudhir-Yadav et al., 2017) and uncertain because of its dependency on monsoon and poor management practices, which result in negative net returns, most often in years of natural calamities such as drought, flood, and cyclone (Barah and Pandey, 2005).

In Odisha State of Eastern India, manual puddled transplanting (PTR) and *beushening* are the most dominant rice establishment methods. *Beushening* is an age-old practice that consists of broadcasting a high seed rate (∼100 kg ha^−1^) prior to the onset of monsoon rain followed by cross ploughing (*beushening* operation) in the standing rice crop 4–6 weeks after rice emergence when about 10 cm of rainwater accumulates, along with *kellua* (redistribution of rice seedlings and removal of uprooted weeds). Seedling redistribution and manual weeding operations are predominantly handled by women farmers/laborers. These operations are rainfall dependent and, if they become delayed, risks of yield losses due to weed competition are much higher in *beushening*. Similarly, for PTR, farmers need to rely on rains or irrigation to accumulate 15–20 cm of standing water for churning of the soil. Both these methods are followed to control weeds and optimize plant stand. However, these methods consume large amounts of labor, energy (for intensive tillage), and water, which are becoming scarce; hence, these traditional crop establishment methods are becoming less attractive and economical. The reported amount of irrigation water required for puddling varies from 100 mm (Sudhir-Yadav et al., 2011) to 544 mm (Bhuiyan et al., 1995). Also, high labor demand at transplanting time and rising labor scarcity and wages are compelling farmers to shift to labor-efficient methods (Kumar and Ladha, 2011). Moreover, semi-dwarf and short-duration cultivars are considered not suitable for *beushening* since stem breakage may occur during cross-ploughing (Gautam et al., 2013). Hence traditional long duration and tall cultivars are used both in PTR and *beushening* which occupies the field for long time.

Dry drill-seeded rice (DSR) is a feasible option to *beushening* and PTR that brings an opportunity to reduce input cost and irrigation water (Dawe, 2005, Humphreys et al., 2005) by using pre-monsoon rainfall more efficiently for crop establishment and early crop growth. The labor requirement in direct seeding is about 34% that of transplanted rice (Ho Nai Kin and Romli, 2002). Balasubramanian and Hill (2000) reported that direct seeding offers a higher tolerance of water deficit and higher profit in areas with an assured water supply. A few other advantages are minimal disturbance of soil structure in DSR and reduction in methane emissions as compared to puddling. Another option to reduce water input is transplanting in non-puddled soil (Malik et al., 2011). Non puddled transplanted rice (NPTR) also helps in timely transplanting, saving energy and therefore input cost (Haque et al., 2015). Input water savings of 35–57% have been reported for dry direct-seeded rice sown into non-puddled soil compared with puddled soil (Sharma et al., 2002, Singh et al., 2002).

Rice, being a staple crop, is grown with utmost importance but with a misconception that it requires an enormous amount of water. High seepage and percolation losses are the main reasons for the much higher water use in flooded rice than in other crops. Improved non-puddled crop establishment has potential to save irrigation water, but water consumption will depend on irrigation scheduling. In clay-loam soil, Sudhir-Yadav et al. (2011) reported higher irrigation input in DSR than in PTR when irrigation was applied daily, whereas ∼33% savings occurred when irrigation was applied at 20 kPa. The response to irrigation schedule and crop establishment method can vary with cultivars. In recent times, public organizations and the private sector have released a few very promising drought-tolerant rice varieties that can produce similar yield under normal conditions but can give higher yield under water stress conditions (Dar et al., 2014).

The performance of cultivars with different duration varies with water stress: some are susceptible during the vegetative stage and some at flowering and reproductive stage (Pantuwan et al., 2002). Lalat is a local variety of Odisha developed by Orissa University of Agriculture and Technology (OUAT). It has medium duration (125–130 days) and is commonly grown in most parts of Odisha. Sahbhagi Dhan is a drought-tolerant and short-duration cultivar (110–115 days) recommended for the eastern states of India developed by the Centre for Rainfed Upland Rice Research Station (CRURRS), Hazaribag, in collaboration with the International Rice Research Institute (IRRI), Philippines. Sahbhagi Dhan has shown a yield advantage of 0.8–1 t ha^−1^ over other varieties under drought conditions (Yamano et al., 2013). Some recently released hybrids (Arize^®^ 6129, Arize^®^ 6444, Arize^®^ Tej, and Arize^®^ Dhani) developed by Bayer are known to perform better in Northern India and parts of Eastern India. Arize^®^ 6444 (135–140 days) and Arize^®^ 6129 (115–120 days) are suitable for the direct-seeded system and for non-puddled mechanically transplanted rice (CSISA Annual Report, 2015), are resistant to bacterial leaf blight (BLB), and produce maximum productive tillers and hence give higher yield. US 323 developed by US Agriseeds is of 115–120 days’ duration with better tillering ability and higher yield.

Therefore, this study was undertaken to evaluate crop growth and development, yield, and irrigation input and understand the trade-off for different cultivars, water regimes, and establishment methods.

## 2 Materials and methods

### 2.1 Experimental site

A replicated field experiment was conducted for two years (2014–2015) and four seasons at the research farm of Orissa University of Agriculture and Technology (OUAT), Bhubaneswar (20°15′N, 85°48′ E, 30.6 m ASL), during the dry season (January to May) and wet season (June to October). The region is characterized by a subtropical climate with average annual rainfall of 1451 mm (Odisha Agriculture Statistics, 2013–14), of which the maximum is received during June to October. The soil at the experimental site is sandy clay loam with uniform texture up to a depth of 100 cm with the exception of the top 10 cm (loam). Selected soil properties at the time of sowing the first crop are shown in Table 1. The last five years’ cropping history was rice in the wet season followed by an oilseed crop such as mustard/groundnut in the dry season.

**Table 1:** Basic soil properties.

Depth (cm)	pH	EC (dS m^−1^)	OC (g/kg)	N (kg ha^−1^)	P (kg ha^−1^)	K (kg ha^−1^)	Texture	Sand (%)	Clay (%)	Silt (%)	BD (g cm^−3^)
0–10	6.4	0.18	4.8	143.8	38.5	117.0	loam	47.6	24.4	28.0	1.67
10–20	6.3	0.14	3.3	137.5	33.2	98.6	sandy clay loam	67.6	25.8	6.60	1.87
20–30	6.5	0.01	4.7	131.3	25.4	125.4	sandy loam	76.6	17.8	5.60	1.88
30–60	7.3	0.10	2.9	62.5	15.8	153.4	sandy loam	75.2	19.1	5.70	1.48

### 2.2 Experimental design

The experimental design was a three-factorial experiment with two crop establishment methods, dry-seeded rice (DSR) and non-puddled transplanted rice (NPTR), three water regimes based on soil water tension (no stress, 10 kPa, and 40 kPa) at 15-cm soil depth, and five cultivars (Lalat, Sahbhagi Dhan, Arize^®^ 6129, Arize^®^ 6444, and US 323), replicated thrice. Thus, there were a total of 30 treatment combinations. The establishment methods (EM) were allotted to the main plot, water regimes (W) to subplots, and cultivars (V) to sub-subplots in a split–split plot design. Each sub-subplot was of 7 m × 4 m size.

### 2.3 Field preparation

The experimental field was cultivated and laser leveled about 2 weeks before the establishment of the experiment followed by the layout of the experiment. Since the water regime treatments were allotted to subplots, each subplot block was bounded by earthen bunds with a plastic lining to a depth of 40 cm to minimize lateral movement of water into and through the bunds. There were 1-m-wide buffers also between all subplots.

### 2.4 Crop management

The seeds of all varieties were treated with Bavistin at 3 g/kg. DSR was sown into dry cultivated soil (10% moisture) by drilling the seed at a depth of 2–3 cm using 30 kg ha^−1^ seed rate with a row spacing of 20 cm. On the same day, the seed/nursery bed for transplanted treatments was sown. For NPTR, a well-prepared field was soaked a day before transplanting to soften the soil to facilitate transplanting. The seedlings (15–20 days old) were transplanted manually in the main field at a row spacing of 23 cm and plant spacing of 14 cm (mimic of mechanical transplanting geometry). Basal application of nitrogen (N) at 18 kg ha^−1^, phosphorus (P_2_O_5_) at 46 kg ha^−1^, potash (K_2_O) at 40 kg ha^−1^, and zinc sulfate at 5 kg ha^−1^ was done using diammonium phosphate (DAP) and muriate of potash (MOP). The basal fertilizers were broadcast before sowing (DSR) or final cultivation (NPTR). Additional 132 kg N ha^−1^ in form of urea was applied in four topdressings at fortnight intervals with the first topdressing at active tillering stage and the last topdressing coinciding with the panicle initiation stage.

#### 2.4.1 Dry season 2014 and 2015

DSR was sown on 8 January 2014 and 2015 whereas NPTR was transplanted on 24 January 2014 and 30 January 2015. Overall germination was good in DSR but in certain spots, gap filling and thinning were done 14 days after sowing (DAS) to maintain a uniform plant stand. Weeds in DSR were controlled by applying a pre-emergence herbicide (pendimethalin at 1000 g a.i. ha^−1^) at 2 DAS and post-emergence herbicide (bispyribac-sodium at 25 g a.i. ha^−1^) at around 25 DAS. In NPTR, weeds were controlled with pretilachlor at 500 g a.i. ha^−1^ at 1 day after transplanting (DAT) and bispyribac at 25 g a.i. ha^−1^ at 12 DAT. Weeds that escaped these treatments were removed manually at 45 DAS/T. After basal application of fertilizer, first, second, and third topdressings of nitrogen at 33 kg ha^−1^ were done at 2-wk intervals starting at 19 DAS in year 1 and at 28 DAS in year 2 and at 24 and 28 DAT in NPTR in years 1 and 2, respectively. The last topdressing was done at panicle initiation (PI) stage, which differed for different cultivars.

In the first season, Lalat was infested with blast. As per visual scoring, the percentage of blast incidence surpassed 75%, and was effectively controlled by spraying tricyclazole 75WP at 0.6 g/L. Sahbhagi Dhan was infested with bacterial leaf blight and was controlled by spraying a mixture of propiconazole at 600 g/acre with streptomycin at 6 g/acre. The crop remained disease free in all treatments in second year.

#### 2.4.2 Wet season 2014 and 2015

After completion of the dry season, the field was prepared for the wet season with light tillage operation. DSR was sown on 13 June 2014 and 7 July 2015, whereas NPTR was planted on 27 June 2014 and 23 July 2015. Apart from basal fertilizer, a top dressing of urea was applied at 15–20 days’ interval starting from 15 to 20 DAS or 23–27 DAT for DSR and NPTR, respectively. Weeds were controlled with the same herbicides followed by one spot handweeding as used in the dry season. Stem borer infestation was observed in both the years, and it was controlled by applying chloropyriphos (50% EC) + cypermethrin (5% EC).

### 2.5 Observations

#### 2.5.1 Irrigation input

Irrigation water was applied using polyvinyl chloride pipes of 10-cm diameter with a separate outlet for each plot. The volume of irrigation applied to each plot was measured using a HWE Woltman type flow meter. For each irrigation cycle, the plots were topped up to 50-mm standing water depth measured with a ruler installed in each plot. Irrigation was scheduled based on the average reading of tensiometer established in subplots. The quantity of water applied and the actual depth of irrigation were computed by dividing the volume of irrigation by the area of the plot.

#### 2.5.2 Soil water tension

Soil water tension (SWT) was measured using a gauge tensiometer (Irrometer^®^) installed in each subplot placed at a depth of 15 cm. SWT was observed every morning around 0800 to determine the need for irrigation on that day.

#### 2.5.3 Crop performance

Plant density (the number of rice plants m^−2^) was determined at 15 DAS for DSR. It was estimated by counting the number of plants from a 0.24-m^2^ area [0.4 m (two rows 20-cm apart) × 60 cm length] from two random locations per plot. Similarly, periodic tiller density was determined from two consecutive rows of 60-cm length (in DSR) or four hills (equivalent to 56-cm row length) at two fixed places in each plot (in NPTR). Tiller count was done at 15 days’ interval commencing from 30 days till flowering. Leaf area index (LAI) was measured by using Accu PAR (LP-80 Ceptometer^®^) on a fortnightly basis from five different spots in a plot with the probe parallel to the rows starting from 30 DAS. Periodic biomass sampling was done by cutting the rice plants at ground level from 0.2576-m^2^ area [0.46 m (two rows with 23-cm spacing) × 0.56-m length (4 hills with 14-cm spacing)] for NPTR and 0.24-m^2^ area (0.4 m × 0.6 m) for DSR from two locations in each plot. The plant samples were oven-dried at 60 °C for 72 h to measure biomass accumulation.

To determine effective tillers, a sample was collected from 60-cm row length from four locations in each plot. Randomly, 20 panicles were selected to determine the number of grains per panicle and filled and unfilled grains per panicle. The dry weight of filled grain was determined by putting it in an oven at 60 °C for 72 h, and the average grain weight was calculated. Floret fertility was calculated as the percentage of filled grain to the total number of florets per panicle.

Grain yield was determined by harvesting the plant sample manually from the center of the plot from an area of 6 m^2^ (3 m × 2 m, i.e., 10 rows to the length of 3 m) in DSR and 6 m^2^ (2.94 m × 2.07 m, i.e., 9 rows to the length of 2.94 m) in NPTR. Grain moisture content was measured for each plot using a moisture meter, and grain yield was expressed as t ha^−1^ at 14% moisture content.

### 2.6 Statistical analysis

All data were analyzed by analysis of variance (ANOVA) using STAR 2.0.1. Treatment means were compared by the least significant difference (LSD) at 5% probability (p = 0.05).

## 3 Results

### 3.1 Weather

**Fig. 1:**
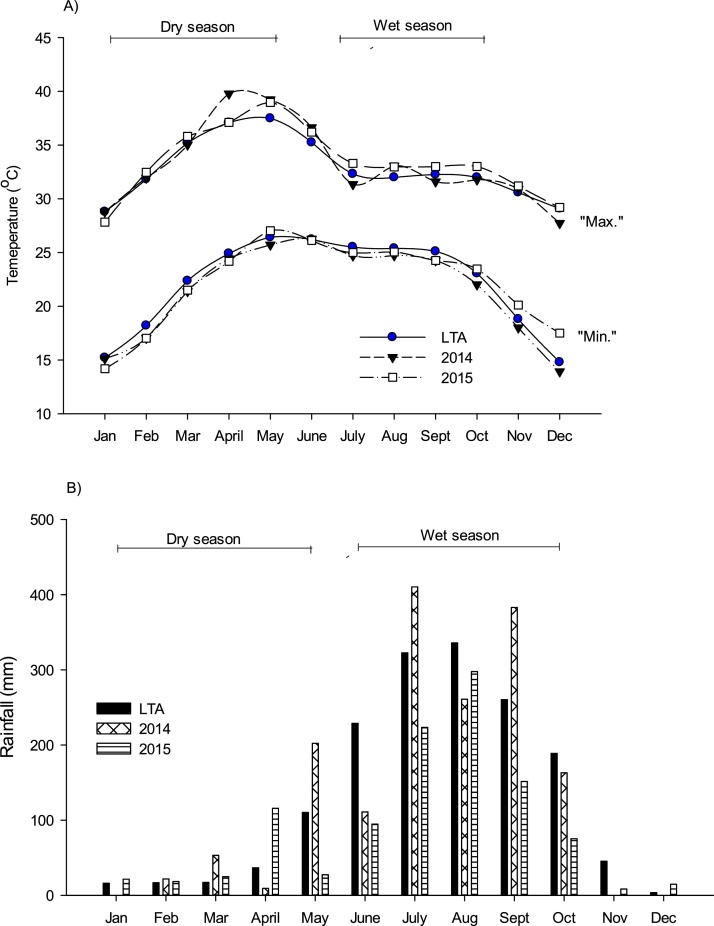
Monthly mean maximum and minimum temperature (A) and monthly total rainfall (b) at Bhubaneshwar, Odisha, in 2014 and 2015, compared with long-term averages (1985 to 2014).

In the dry season (both 2014 and 2015), the monthly average maximum (T max.) and minimum temperature (T min.) were similar to the long-term average (average of dry season 1985–2014), which was 34.1 and 21.4 °C, respectively (Fig. 1). The total amount of rainfall received during this period in 2014 and 2015 was 89 and 11 mm more than the long-term average (LTA), respectively. However, the number of rainy days in both 2014 (11 days) and 2015 (17 days) was less than the long-term average (24 days).

In the wet season, the rainfall in 2014 was 1329 mm, which was similar to the LTA (1337 mm). However, in 2015, the rainfall (843 mm) was significantly less than the LTA. The number of rainy days in both 2014 (84) and 2015 (66) was less than the LTA (125 days). The rainfall pattern from June to October was similar in both years, with maximum accumulation in July-September.

### 3.2 Irrigation

#### 3.2.1 Dry season

The average amount of irrigation applied was similar in both years (678 mm in 2014 and 681 mm in 2015). The irrigation input was similar in DSR and NPTR in 2014; however, in the second year, the irrigation input was 48 mm (7%) higher in DSR than in NPTR (Table 2).

**Table 2:** Water input and productivity under different establishment methods, water regimes, and cultivars.

	Irrigation (mm)				Irrigation water productivity (g kg^−1^)				Input water productivity (g kg^−1^)			
	Dry season		Wet season		Dry season		Wet season		Dry season		Wet season	
	2014	2015	2014	2015	2014	2015	2014	2015	2014	2015	2014	2015
Establishment method (EM)												
DSR	695	705	272	245	0.90	0.93	2.06	2.15	0.80	0.73	0.34	0.49
NPTR	660	657	206	197	0.94	0.99	3.13	2.97	0.82	0.73	0.38	0.52
LSD (p ≤ 0.05)	NS	28.7	5.29	12.7	NS	NS	0.16	0.34	NS	NS	0.02	NS

Water regimes (W)												
No stress	911	972	342	346	0.78	0.70	1.61	1.39	0.71	0.61	0.34	0.44
10 kPa	663	742	241	180	0.95	0.82	2.16	2.70	0.84	0.68	0.36	0.51
40 kPa	458	328	134	138	1.04	1.35	4.01	3.59	0.87	0.92	0.39	0.55
LSD (p ≤ 0.05)	53.8	12.4	5.72	6.73	0.11	0.06	0.15	0.32	0.09	0.04	0.03	0.05

Varieties (V)												
Lalat	679	674	245	220	0.79	1.00	2.14	2.24	0.70	0.76	0.31	0.43
Sahbhagi Dhan	641	665	173	192	0.89	0.82	2.91	2.69	0.78	0.67	0.36	0.49
Arize^®^ 6129	690	688	255	205	0.91	0.95	2.70	2.93	0.80	0.72	0.39	0.56
US 323	688	688	253	218	0.99	0.96	2.20	2.49	0.86	0.73	0.31	0.49
Arize^®^ 6444	690	688	268	271	1.03	1.05	3.02	2.45	0.90	0.79	0.44	0.52
LSD (p ≤ 0.05)	15.0	5.37	8.15	5.83	0.08	0.06	0.22	0.24	0.07	0.05	0.02	0.04

Interaction												
EM × W	NS	NS	9.98	NS	NS	NS	0.27	0.59	NS	NS	NS	NS
EM × V	NS	NS	13.7	16.7	NS	NS	038	NS	NS	NS	NS	NS
W × V	NS	51.1	70.7	52.0	NS	NS	1.90	2.13	NS	NS	NS	NS
EM × W × V	NS	NS	20.0	14.3	NS	NS	NS	NS	NS	NS	NS	NS

In both years, water regime significantly influenced irrigation application, and irrigation water decreased in the following order: no stress (912–972 mm) > 10 kPa (663–742 mm) > 40 kPa (328–458 mm) (Table 2). When irrigation was applied at 10 kPa and 40 kPa SWT, irrigation water application decreased by 239 mm (25%) and 549 mm (58%) compared with no stress treatment, respectively.

Rice cultivars also significantly influenced irrigation water use (Table 2). Sahbhagi Dhan required the lowest irrigation water among the other cultivars tested in this study. Also, the other cultivars did not differ among themselves in terms of irrigation water use. Additionally, the water regime effect on irrigation water application also differed with rice cultivars as evidenced by significant water regime × rice cultivar interaction in 2015. The irrigation input was significantly less in Sahbhagi Dhan in no stress treatment than in other cultivars.

**Fig. 2:**
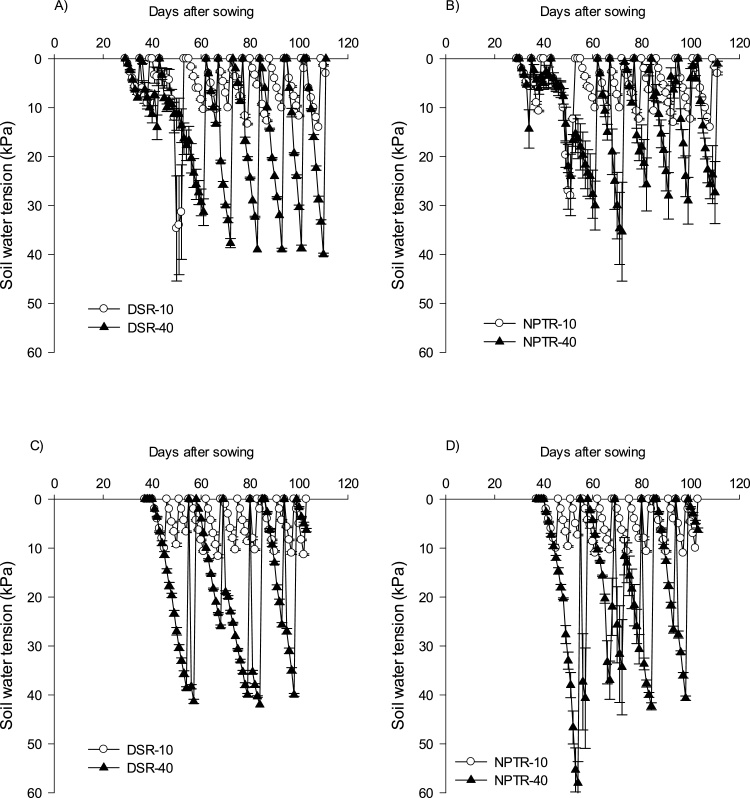

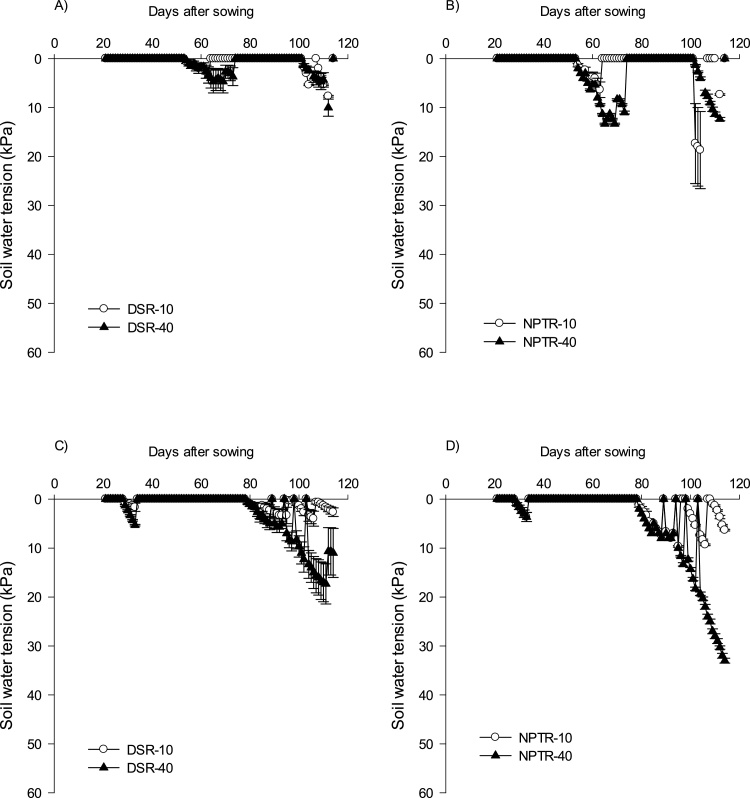
(a) Soil water tension at 15-cm depth in the dry season in 2014 (A–B) and 2015 (C–D). (b) Soil water tension at 15-cm depth in the wet season in 2014 (A–B) and 2015 (C–D).

The water regime treatments were started 25 days after seeding in DSR and 10 days after transplanting in NPTR. In 2014, the first irrigation in 10 and 40 kPa SWT was applied at 38 and 61 DAS, whereas, in the second year, it became further delayed to 43 DAS in 10 kPa and to 57 DAS in 40 kPa. After commencement of the irrigation treatment, the number of irrigations to DSR and NPTR was similar. With 10 kPa SWT, the number of irrigations was 9 in DSR and in NPTR (in 2014), whereas, in 2015, it was 10. With higher water stress of 40 kPa, the number of irrigations decreased to 6 in 2014 and 2 in 2015 in both DSR and NPTR (Fig. 2a & b ).

#### 3.2.2 Wet season

In the wet season, the soil mostly remained saturated due to well-distributed rainfall throughout the season. In both years, there were no sufficiently long dry spells to achieve 40 kPa SWT throughout the season. The first cycle for 10 kPa SWT commenced at 63 DAS in 2014 and at 95 DAS in 2015. After commencement of stress, a total of 3 irrigations were given for 10 kPa SWT in 2014 and 2 irrigations in 2015.

During the wet season, irrigation water application was 31.5% and 24.2% higher in 2014 and 2015, respectively, in DSR than in NPTR (Table 2). Similar to the dry season, in both years, irrigation water application declined with an increase in water stress treatments. As compared to no water stress, irrigation water use declined by 29–48% at 10 kPa and about 60% in 40 kPa water regime. Among cultivars, during both years, irrigation water use was lower in shorter duration cultivars (Lalat, Sahbhagi Dhan, Arize^®^ 6129, US 323) than in medium-duration cultivar Arize^®^ 6444.

Significant interaction between establishment method with cultivar and water regimes with cultivar was observed in both the years. Highest irrigation was applied in Arize® 6444 under DSR under no stress condition.

### 3.3 Crop performance

#### 3.3.1 Dry season

The plant stand in DSR was quite good in both years, with an average plant density of 130 plants m^−2^. The plant density was significantly higher in 2014 (147 plants m^−2^) than in 2015 (114 plants m^−2^). In NPTR, plant density was the same in all varieties in both years.

**Fig. 3:**
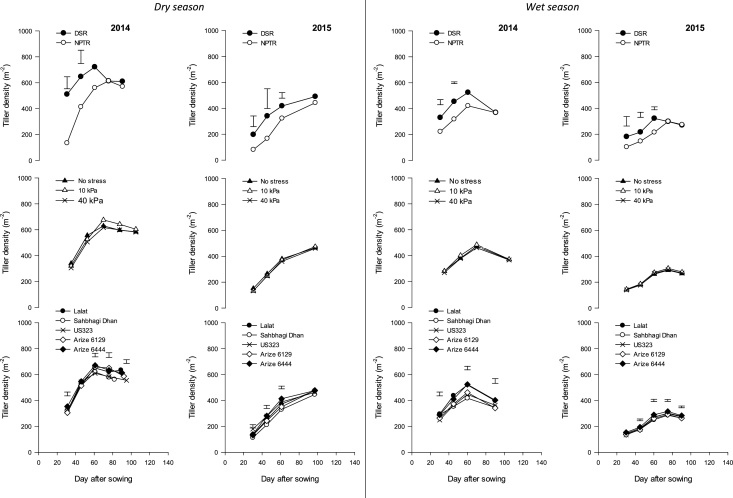
Effect of rice establishment method, water regimes, and cultivars on tiller density at different growth stages in dry and wet seasons in 2014 and 2015. Vertical bars indicate LSD 0.05 for comparing all treatment combinations.

**Fig. 4:**
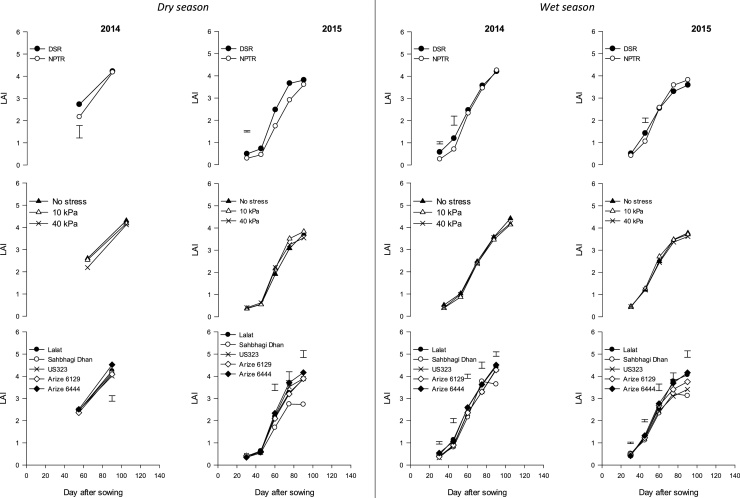
Effect of rice establishment method, water regimes, and cultivars on leaf area index (LAI) at different growth stages in dry and wet seasons in 2014 and 2015. Vertical bars indicate LSD 0.05 for comparing all treatment combinations.

Tiller density increased up to 60 DAS in DSR and then declined, but, in NPTR, maximum tiller density was observed at 75 DAS (Fig. 3). Tiller density was significantly higher with DSR at an early stage (up to 45 DAS in 2014 and 61 DAS in 2015). There was no effect of water management on tiller density. Tiller density was highest with Arize^®^ 6444 at almost all stages (except 97 DAS in 2015). LAI was significantly affected by cultivars, and the highest LAI (at 90 DAS) was observed in Arize^®^ 6444 in both 2014 (4.52) and 2015 (4.17) (Fig. 4). LAI for all cultivars increased till 90 DAS and then declined except in Sahbhagi Dhan, in which it declined after 75 days. In both years, there was no significant effect of water stress on LAI in establishment methods.

**Fig. 5:**
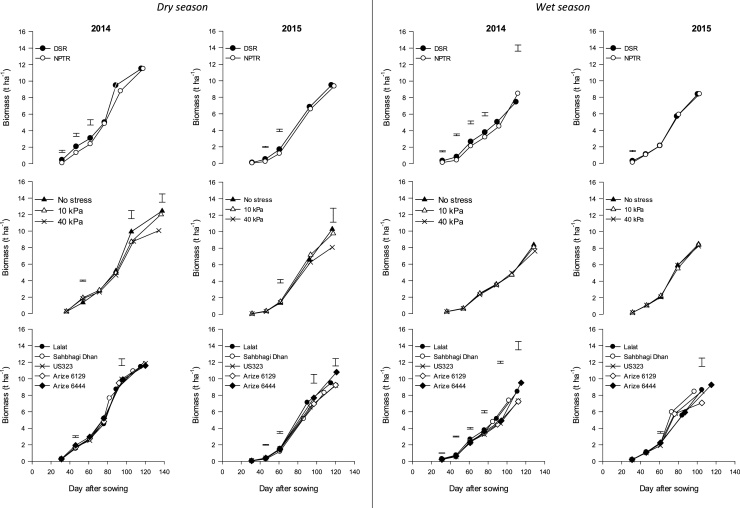
Effect of rice establishment method, water regimes, and cultivars on biomass accumulation at different growth stages in dry and wet seasons in 2014 and 2015. Vertical bars indicate LSD 0.05 for comparing all treatment combinations.

In general, biomass accumulation was higher in 2014 than in 2015. The average biomass in 2014 was 11.5 t ha^−1^ while it declined to 9.4 t ha^−1^ in 2015. Among treatments, DSR had significantly higher biomass than NPTR up to 60 DAS in both years (except at 31 DAS in 2015) as shown in Fig. 5. Water management has a significant effect on biomass, especially at flowering and physiological maturity. Biomass accumulation was significantly higher in the no stress treatment than in 40 kPa but on a par with 10 kPa. There was a 19% and 22% decline in biomass with 40 kPa vis-à-vis no stress in treatments in 2014 and 2015, respectively. Among cultivars, Arize^®^ 6444 had significantly higher biomass at an early stage than all other cultivars in the first season and higher than Sahbhagi Dhan and Lalat in 2015. In 2014, the biomass of all cultivars was similar at physiological maturity, but, in 2015, Arize^®^ 6444 produced significantly higher biomass at all stages.

There was an interaction of establishment method and cultivars at the early stage of crop growth (45 DAS in 2014 and 30 DAS in 2015). In 2014, biomass accumulation was significantly higher in DSR in all the cultivars, with the highest in Arize^®^ 6444. In 2015, biomass accumulation in DSR was significantly higher in US 323 and Arize^®^ 6444 than in NPTR. In 2015, a significant interaction of establishment method and water stress was observed at 60 DAS and PM stage. At 60 DAS, biomass accumulation was higher at 10 kPa in DSR than in NPTR.

#### 3.3.2 Wet season

The average plant stand in DSR was similar (97 plants m^−2^) in both years. The cultivars varied significantly across the years, with the highest plant stand in Arize^®^ 6444. In NPTR, plant density remained the same in all varieties in both years.

There was a significant effect of establishment method on tiller density till 45 DAS in 2014 and until 60 DAS in 2015 (Fig. 3). Tiller density for DSR increased up to 60 DAS while in NPTR the maximum tiller density was recorded at 75 DAS and thereafter declined. Among cultivars, a significantly higher tiller density was attained in Arize^®^ 6444 in both years except at 45 DAS in the first year, when Lalat had more tillers. At early stage (30 and 45 DAS), there was also an interaction of establishment method and cultivars on tiller density in 2015. The tiller density of Sahbhagi Dhan was significantly less with NPTR than other cultivars LAI was significantly affected by cultivars at all stages in both years, but without any consistent trend (Fig. 4). Significantly higher LAI of DSR over NPTR was recorded at 30 and 45 DAS in 2014 and randomly at 45 DAS in 2015. There was no effect of water regimes on LAI.

Biomass accumulation was higher in 2015 (8.39 t ha^−1^) than in 2014 (7.99 t ha^−1^) as depicted in Fig. 5. Biomass accumulation was significantly affected by establishment method at almost all stages in 2014, but, in 2015, it was significant only at 30 DAS. The biomass of DSR was significantly greater than that of NPTR till 75 DAS in 2014. In both years, biomass accumulation was highest in NPTR at PM stage. Water regimes had no significant effect on biomass accumulation in both years. Cultivars showed a significant effect at all stages in 2014 but only at 60 DAS and PM in 2015. The highest biomass accumulation was attained by Arize^®^ 6444 in 2014 (9.51 t ha^−1^) and 2015 (9.26 t ha^−1^).

### 3.4 Yield attributes and yield

#### 3.4.1 Dry season

There was a significant interaction of establishment method, water regime, and cultivars on fertile tillers m^−2^ in 2014 (Table 3). Within DSR, fertile tillers were similar in all water regimes with Arize^®^ 6444, US 323, and Lalat, while Arize^®^ 6129 and Sabhaghi Dhan had more fertile tillers at 10 kPa than at no stress. However, with NPTR, this trend was seen only with Arize^®^ 6444. Within irrigation regimes, there was no significant difference in fertile tiller density at no stress, but there were more tillers in DSR with Arize^®^ 6129 at 10 kPa and Lalat at 40 kPa. In 2015, there was no interaction among any treatments. However, fertile tiller density was significantly higher in DSR (470) than in NPTR (409). Among water regimes, fertile tiller density was significantly higher with 10 kPa. Fertile tillers were similar in all cultivars. Filled grains panicle^−1^ indicated an interaction effect of establishment method and cultivars. Filled grains panicle^−1^ were significantly higher in NPTR with all cultivars except US 323, which had more filled grains panicle^−1^ in DSR. Among water regimes, filled grains panicle^−1^ varied from 47 to 93 and were significantly higher in no stress and 10 kPa treatments. In the second year, NPTR produced significant higher filled grains panicle^−1^ than DSR. There was a significant interaction of water regime and cultivars. Arize^®^ 6129 and 6444 produced maximum filled grains panicle^−1^, which was similar to other cultivars and water regimes except at no stress, when it was significantly higher than Lalat and Sahbhagi Dhan.

**Table 3:** Grain yield and yield attributes of different cultivars under different establishment methods and water regimes.

	Effective tillers				Filled grains panicle^−1^				Average grain weight (mg)				Floret fertility (%)				Grain yield (t ha^−1^)			
	Dry season		Wet season		Dry season		Wet season		Dry season		Wet season		Dry season		Wet season		Dry season		Wet season	
	2014	2015	2014	2015	2014	2015	2014	2015	2014	2015	2014	2015	2014	2015	2014	2015	2014	2015	2014	2015
Establishment method (EM)																				
DSR	562	460	315	285	78	54	78	70	19.8	20.7	20.3	21.9	74.7	71.5	80.1	78.9	6.04	5.87	4.91	4.66
NPTR	472	409	307	267	85	58	79	74	20.3	20.8	20.7	21.0	71.0	71.9	77.3	77.3	6.01	5.68	5.19	4.75
LSD (p ≤0.05)	53.7	NS	NS	NS	NS	3.49	NS	NS	NS	NS	NS	0.60	NS	NS	NS	NS	NS	NS	0.27	NS

Water regimes (W)																				
No stress	506	433	311	273	93	63	79	71	22.0	22.3	20.4	21.5	75.2	74.2	79.0	77.0	7.09	6.81	5.15	4.72
10 kPa	537	466	322	281	85	57	81	71	19.9	20.3	20.5	21.4	75.5	72.9	79.9	77.8	6.24	6.11	5.00	4.64
40 kPa	508	404	299	274	67	47	75	73	18.2	19.6	20.7	21.6	67.9	68.0	77.1	79.4	4.74	4.41	5.01	4.74
LSD (p ≤ 0.05)	NS	39.7	NS	NS	9.7	4.0	NS	NS	1.1	1.4	NS	NS	NS	4.4	NS	NS	0.42	0.28	NS	NS

Varieties (V)																				
Lalat	619	468	388	302	67	49	79	60	19.7	20.9	19.7	21.3	68.1	69.5	79.7	75.0	5.18	5.97	4.35	4.12
Sahbhagi Dhan	513	428	271	270	73	55	72	74	19.9	19.9	20.5	21.9	72.3	73.7	78.6	79.7	5.42	4.97	4.71	4.40
Arize^®^ 6129	494	426	280	263	97	57	78	72	19.5	20.9	20.8	20.5	78.0	74.0	77.0	78.0	6.09	5.75	5.52	5.16
US 323	453	418	288	254	92	60	67	70	19.4	20.2	20.6	21.5	71.4	70.7	75.6	77.3	6.56	5.78	4.42	4.59
Arize^®^ 6444	507	432	328	292	78	58	96	84	21.8	21.9	21.1	22.3	74.3	70.2	82.1	80.5	6.86	6.41	6.28	5.24
LSD (p ≤ 0.05)	31.3	34.0	25.9	18.2	8.0	5.4	7.4	6.5	1.7	1.2	NS	NS	4.7	NS	3.8	3.3	0.50	0.30	0.34	0.33

Interaction																				
EM × W	NS	NS	NS	NS	NS	NS	12.8	12.2	NS	NS	NS	NS	NS	NS	NS	NS	NS	NS	NS	NS
EM × V	76.1	NS	NS	46.0	23.2	NS	NS	NS	NS	NS	NS	NS	13.7	NS	NS	NS	NS	NS	NS	NS
W × V	NS	NS	NS	NS	NS	NS	NS	NS	NS	NS	9.4	NS	NS	NS	NS	NS	NS	NS	NS	NS
EM × W × V	76.6	NS	NS	NS	NS	NS	NS	NS	4.1	NS	NS	NS	NS	NS	NS	NS	NS	NS	NS	NS

A significant interaction of establishment method, water regime, and cultivars on average grain weight was observed in 2014. Within DSR, Sahbhagi Dhan produced similar average grain weight in all water regimes, but, with other cultivars, grain weight declined significantly at either 10 kPa or 40 kPa. With NPTR, Sahbhagi Dhan, US 323, and Arize^®^ 6444 had similar grain weight in all water regimes. In 2015, there was no interaction, but average grain weight was significantly higher with no stress water regime (22.4 mg). Among cultivars, Arize^®^ 6444 resulted in maximum average grain weight (21.9 mg).

Grain yield in 2014 (6.03 t ha^−1^) was significantly higher than in 2015 (5.78 t ha^−1^). In both years, grain yield was similar with DSR and NPTR but was significantly affected by irrigation schedule and cultivars. Grain yield with no water stress was 7.1 and 6.8 t ha^−1^ in 2014 and 2015, respectively, which was significantly higher than at 10 and 40 kPa. The average yield penalty with 10 kPa and 40 kPa compared with no stress treatment was 12% and 35%, respectively. Among cultivars, Arize^®^ 6444 produced the highest yield in both years. The grain yield of Arize^®^ 6444 was similar to that of US 323 (6.7 t ha^−1^) in the first year but was significantly higher than that of all varieties in 2015. The gain in yield with varieties varied from 22% to 24% in two dry seasons.

#### 3.4.2 Wet season

There was no significant effect of establishment method and water regime on effective tillers and filled grains in both years. Average grain weight was significantly affected by establishment method in 2015, and the grain weight of DSR was 4.1% higher than that of NPTR. Effective tillers and filled grains panicle^−1^ were significantly affected by cultivars in both years. The highest number of effective tillers was recorded in Lalat with 388 (2014) and 302 (2015). Filled grains panicle^−1^ were highest in Arize^®^ 6444 in both 2014 (96) and 2015 (84). In both years, there was a significant interaction between establishment method and water stress in filled grains panicle^−1^ and the highest was found in NPTR under 10 kPa. In the first year, there was a significant interaction between water stress and cultivars in average grain weight, with the highest attained by Arize^®^ 6444 under 10 kPa.

Grain yield was much lower in 2015 (4.7 t ha^−1^) than in 2014 (5.05 t ha^−1^) (Table 3). There was a significant effect of establishment method on grain yield in 2014. The yield of NPTR surpassed that of DSR by 5.7% (2014) and 1.9% (2015). There was no effect of water regimes on grain yield. Grain yield was significantly affected by cultivars in both years. Higher grain yield was produced by Arize^®^ 6444 in 2014 (6.28 t ha^−1^) than in 2015 (5.24 t ha^−1^).

### 3.5 Water productivity

#### 3.5.1 Dry season

There was a significant effect of water regimes and cultivar on water productivity in both years (Table 2). Among water regimes, irrigation water productivity (WP_I_) increased significantly with alternate wetting and drying at 10 kPa and 40 kPa than no stress treatment. In 2014, WP_I_ with 40 kPa was 9% and 33% higher than 10 kPa and no stress while in 2015; it further increased to 65% and 93%, respectively. Among cultivars, WP_I_ was highest with Arize^®^6444 in both years

Input water productivity (WP_I+R_) had a similar trend as WP_I_. WP_I+R_ was significantly higher with 40 kPa i.e. 0.8 g kg^−1^ in 2014 and 0.9 g kg^−1^ in 2015. Among the cultivars, Arize^®^ 6444 showed the highest WP_I+R_ with 0.9 g kg^−1^ in 2014 and 0.7 g kg^−1^ in 2015.

#### 3.5.2 Wet season

All the treatments showed a significant effect on water productivity in both years except on establishment in 2015 (Table 2). Irrigation water productivity (WP_I_) was significantly higher in NPTR in both years. With the increase in water stress, WP_I_ was increased, with a maximum at 40 kPa SWT. Among cultivars, the highest WP_I_ was obtained by Arize^®^ 6444 in 2014 and Arize^®^ 6129 in 2015.

Higher input water productivity (WP_I+R_) was observed in 2015 for all the treatments compared with 2014. It showed a similar trend to that of WP_I_ in both years with maximum productivity with 40 kPa SWT and Arize^®^ 6129.

## 4 Discussion

### 4.1 Yield trends

In general, yield in the dry season was higher than in the wet season, perhaps because of greater bright sunshine hours, which is directly related to the accumulation of photosynthates (Bueno and Lafarge, 2009). Effective tillers were more numerous in the dry season than in the wet season. Similar results were obtained by Sudhir-Yadav et al. (2014).

There was no significant difference in grain yield between establishment methods in two dry seasons. Similar results were obtained by Sudhir-Yadav et al. (2014) at IRRI, Philippines. These results were in contrast to those of Singh et al. (2001), who showed a higher yield of transplanting under non-puddled conditions compared with DSR in a 3-year experiment in sandy loam soil in Delhi, which might be because of better weed control under transplanted conditions. As the threshold for irrigation increased from no stress to 40 kPa, yield declined. This could be explained by reduced chlorophyll content with an increase in stress due to electrolyte leakage and hence a reduction in yield (Petrov et al., 2012, Jahan et al., 2014). Maheswari et al. (2007) found that increasing water stress in rice resulted in many anatomical changes in the plant, such as a decrease in cell volume, a decrease in cell division, a decrease in intercellular spaces, and a thickening of the cell wall, which limit overall plant growth. Among cultivars, higher yield was obtained by Arize^®^ 6444, which could be because of higher average grain weight and more numerous effective tillers. In general, hybrids outperform inbreds in terms of yield (Julfiquar, 2009, Yang et al., 2007), perhaps because of better sink regulation (Lafarge and Bueno, 2009) and higher average grain weight (Huang et al., 2012, Jiang et al., 2016).

The yield trend was similar in the wet season except for overall low yield. Grain yield was significantly higher in Arize^®^ 6444, and yield attributes such as filled grains panicle^−1^ and floret fertility also showed the same feature. Similar results were put forth by Zhang et al. (2012) in the dry and wet seasons at IRRI, Philippines.

### 4.2 Crop performance

Plant stand in DSR was lower in the wet season than in the dry season, which might be linked with rain pattern. On average, it took 5, 8, and 11 days more in the dry season than in the wet season to attain PI, 50% flowering, and PM stage, respectively. Average tiller density and aboveground biomass in the dry season were higher than in the wet season. Similar results were obtained by Sudhir-Yadav et al. (2014).

The performance of DSR in terms of tiller density, development of leaf area, and biomass accumulation was better than that of NPTR in both years, which could be attributed to higher early vigor in DSR and slower early growth of NPTR due to transplanting shock. Root damage during transplanting affects early seedling vigor and also early growth (Ros et al., 2003). Relatively higher growth in DSR than in the transplanted crop is consistent with the findings of many studies (Sudhir-Yadav et al., 2011, Kumar and Ladha, 2011, Tuong et al., 2000). There was no significant difference in terms of water stress on tiller density and LAI. After flowering, aboveground biomass decreased significantly with an increase in stress, reflecting reduced leaf area and growth due to water stress. Similar findings were reported by Bouman and Tuong (2001) and Belder et al. (2004). Cultivars differed significantly in terms of growth parameters.

### 4.3 Water accounting

Total irrigation input in the dry season was roughly more than double that in the wet season irrespective of the treatments, reflecting lower rainfall and higher evaporative demand in the dry season. The amount of rainfall received in the wet season was approximately four times that received in the dry season and the evaporation rate in the dry season was about 0.7 times higher than in the wet season. The amount of irrigation applied in 2015 was higher than in 2014 irrespective of treatments, which could be because of higher rainfall received in 2014. In all the seasons, the amount and number of irrigations applied in DSR was higher than in NPTR. It is often argued that DSR saves irrigation water by eliminating the need to puddle (Gopal et al., 2010). However, in our experiments, the amount of irrigation water applied to DSR from the time of sowing to imposing stress or establishment of the crop was higher than the amount of irrigation applied for irrigating the non-puddled field before transplanting till imposing the first stress. The irrigation interval differed with the stress and so the number of irrigations. However, the irrigation interval to reach 10 kPa in both dry seasons ranged from 6 to 12 days, which is a little bit on the higher side due to hardpan presence underneath, which probably reduced the infiltration rate. Irrigation water productivity was 2.5 times higher in the wet season than in the dry season, which might be because of higher rainfall and lower irrigation requirement. Similar results were obtained by Sudhir-Yadav et al. (2014) at IRRI, Philippines. Input water productivity was higher in the dry season than in the wet season, which could be because of higher irrigation being applied in the dry season.

At all the levels of stress, the amount of irrigation applied in 2015 was greater than in 2014. This could be because the amount of rainfall received in 2015 wet season was 79 mm lower than in 2014. But, the rainfall distribution throughout the season was uniform in 2015, with a higher number of rainy days, which proves it took extra time to reach the particular stress. Hence, even though the amount of irrigation applied in 2015 was higher than in 2014, the number of irrigations was slightly lower. The water productivity of Arize^®^ 6444 was higher in both years, which might be because of higher yield per unit of water applied.

The amount of rainfall received in 2014 dry season was higher than in 2015 by 486 mm. Hence, the amount of irrigation applied in 2015 was greater than in 2014 at all the stress levels except 10 kPa. The number of irrigations applied was nearly the same in both years. The results suggest that WP_I_ and WP_(I+R)_ were higher in NPTR than in DSR, indicating that NPTR can perform well in the wet season.

### 4.4 Trade-off with establishment method, water management, and cultivars

**Fig. 6:**
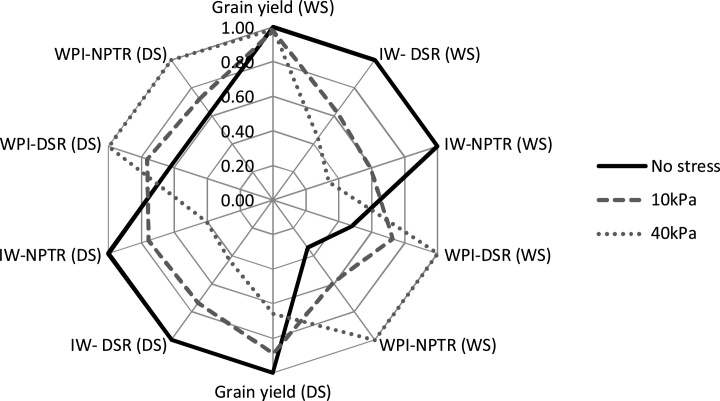
The trade-off with water regimes in terms of grain yield, irrigation water (IW), and irrigation water productivity (WPi) in dry-seeded rice (DSR) and non-puddled transplanted rice (NPTR). DS is dry season and WS is wet season.

In our study, the performance of both DSR and NPTR was similar in all seasons. Within each establishment method, the performance of hybrids (mainly Arize^®^) was better than that of inbreds with and without water stress and therefore the hybrids had high water productivity also; however, the irrigation input was less with short-duration varieties (Sahbhagi Dhan). With water regimes, there was a clear trade-off among yield, irrigation input, and irrigation water productivity with both establishment methods (Fig. 6).

## 5 Conclusions

Many factors can affect the selection of rice establishment method, including climate, season, topography, soil type, labor, water availability, and management skill. In the wet season, which is prominently rainfed, dry seeding can provide the opportunity for the early establishment of the crop before the start of rains. However, there can be a trade-off with supplementary irrigations, which may need to be provided to establish the DSR if a dry spell occurs after seeding. Also, in the region, where heavy rainfall is likely around the time of seeding, DSR may not be a good option because of a lack of anaerobic germination traits in current varieties. In the case of rain occurring before seeding, it is not possible to use seed drills. For these situations, NPTR can be a good option for the establishment of the crop on time, and with a lower water requirement for crop establishment than DSR because it takes 2–3 weeks’ time to grow as a mat nursery in a small area. However, raising a mat nursery needs more skill than seeding the crop directly in the field. Along with age and health of seedlings, transplanting at the right geometry and adjusting the number of seedlings per hill or depth of transplanting require a good knowledge about the machine transplanter. Manual transplanting under non-puddled conditions is more labor demanding besides being very tedious as the soil is generally not so soft.

Among water regimes, there can be significant irrigation savings with mild stress but with some trade-off in grain yield. Many other studies indicate that yield can be maintained at 10–20 kPa, but this will depend on season, the number of dry cycles in a season, and the time of dry cycles in a season.

Among cultivars, hybrids generally outperformed inbreds in both establishment methods and among three water regimes. However, the performance of hybrid US 323 was poor in the wet season. Among hybrids, Arize^®^ 6444 performed better among all cultivars. The main challenges for adoption of hybrids are high seed cost and replacement of the seed in every season. Most farmers generally use their own seed and therefore like to grow inbreds. Sahbhagi Dhan seems to be a good option when there is a high risk of drought.

Further evaluation under a wider range of environmental conditions (e.g. soil texture, climate,) and varieties, is needed to develop irrigation scheduling and other management guidelines for growing rice under non-puddled conditions.
